# Associations between simultaneous use of alcohol and cannabis and cannabis-related problems in 2014–2016: evidence from the Washington panel survey

**DOI:** 10.1186/s42238-024-00217-z

**Published:** 2024-02-24

**Authors:** Yachen Zhu, Yu Ye, Thomas K. Greenfield, William C. Kerr

**Affiliations:** Alcohol Research Group, Public Health Institute, Emeryville, CA 94608 USA

**Keywords:** Simultaneous use of alcohol and marijuana/cannabis, cannabis-related problems, Recreational marijuana legalization, Panel survey, Washington State

## Abstract

**Background:**

To address the research question of how simultaneous users of alcohol and cannabis differ from concurrent users in risk of cannabis use problems after the recreational marijuana legalization in Washington State.

**Methods:**

We used generalized estimating equations with a Poisson distribution to analyze the association between simultaneous use of alcohol and marijuana (SAM) and cannabis-related problems compared to concurrent use. The data is a longitudinal sample of drinkers and cannabis users (*n* = 257, 47% female) aged 18 years and older from Washington State in 2014–2016. We adjusted for survey weights to account for differential probability of selection and response rates. The primary outcome is the past-six-month CUDIT problem subscale (ranging from 0 to 28), which is the total score for seven CUDIT problem items, after excluding the three items that covered marijuana use frequency. Covariates include marijuana use frequency (daily/near daily use, regular use, or infrequent use), marijuana daily quantity, alcohol daily volume, panel survey cycle, medical marijuana recommendation, driving time to nearest marijuana outlet, age of marijuana use onset, and other demographics.

**Results:**

After adjusting for covariates, we found that compared to concurrent use, SAM was significantly positively associated with CUDIT problem subscale (IRR = 1.68, 95% CI: 1.25–2.27, *p* < 0.001); daily/near daily use of marijuana was strongly significantly associated with CUDIT problem subscale compared with infrequent use (IRR = 5.1, 2.71–9.57, *p* < 0.001) or regular use (IRR = 3.05, 1.91–4.85, *p* < 0.001). Secondary analyses using CUDIT total score as the outcome also showed a significant positive association with SAM compared to concurrent use (IRR = 1.17, 1.02–1.34, *p* < 0.05).

**Conclusions:**

This study highlighted the importance of SAM, in addition to cannabis use frequency for predicting cannabis-related problems.

**Supplementary Information:**

The online version contains supplementary material available at 10.1186/s42238-024-00217-z.

## Background

Cannabis use has been associated with a number of long-term health problems, including cognitive decline, major depression, mood and anxiety disorders, and damage to the respiratory, cardiovascular, and reproductive systems (Renard et al. 2013; Campeny et al. 2020; Hasan et al. 2020; Onaemo et al. 2021; Jouanjus et al. 2017). Among various cannabis/marijuana use patterns, simultaneous use of alcohol and marijuana (SAM) at the same occasion can be especially dangerous because multiple drugs can have additive or synergistic effects on the consumer. When people use alcohol and marijuana together, they tend to consume higher quantities and frequencies of both substances compared with when they use the two substances concurrently (use of both substances in general but not necessarily during the same episode) or either substance alone (Brière et al. 2011; Terry-McElrath et al., 2013; Subbaraman and Kerr 2015; Lee et al. 2022; Gonçalves et al. 2021 ). SAM has been shown to be more detrimental than concurrent use or other use patterns, with significantly increased likelihood of alcohol dependence, binge drinking, drunk driving, and other alcohol-related consequences (McCabe et al. 2006; Midanik et al. 2007; Subbaraman and Kerr 2015, 2018). The linkage between SAM and alcohol problems may in part be explained by the Gateway Hypothesis, i.e., people at any higher level of drug use (e.g., cannabis or other illicit drugs) tend to have used all lower-ranked drugs (e.g., alcohol or tobacco) as well, and the progression from low-ranked drugs to high-ranked drugs is strongly associated with the intensity of use at the prior stage (Kandel 1975; Kandel 2002; Kandel and Faust 1975; Donovan and Jessor 1983; Martin et al. 1996). For example, people who use cannabis tend to have also used alcohol, and alcohol problems tend to emerge following the onset of cannabis use (Donovan and Jessor 1983).

The recreational marijuana legalization (RML) in Washington and other US states has resulted in noticeable increases in the prevalence of cannabis use, SAM, and cannabis use disorder in people aged 21 or more years old (Cerdá et al. 2020; Martins et al. 2021; Gonçalves et al. 2022; Zellers et al. 2023). However, little is known about the potential effects of SAM on cannabis-related problems, especially after RML. Before RML, one study reported a significant association between frequent SAM and perceived cannabis dependence in adolescents, although weaker than that with alcohol dependence (Terry-McElrath et al., 2013). Another study among undergraduate college students found that simultaneous polydrug (alcohol and prescription drugs) users were 2 times more likely to report at least three drug use-related problems than concurrent users based on Drug Abuse Screening Test-Short Form (DAST-10); however, this study only investigated the effects of simultaneous use of alcohol and four prescription drugs, which did not include SAM (McCabe et al. 2006). Additionally, both of these two studies were conducted before RML, and focused on adolescents only. Since the RML targeted adults aged 21 years and older, nationwide studies found significant increases in cannabis use, SAM, and cannabis use disorder after the RML in this population (Cerdá et al. 2020; Martins et al. 2021; Gonçalves et al. 2022; Zeller et al. 2023). It has also been reported in Washington State that the age group of 50 + years old significantly increased cannabis use and SAM in 2014–2016 following the enactment of recreational cannabis laws (Subbaraman and Kerr 2020, 2021). With this remarkable change, it is thus important to further investigate the association between SAM and cannabis-related problems in adults after the RML.

In this study, we used a longitudinal adult sample of drinkers and cannabis users drawn from representative samples from Washington State to address the research question: How do simultaneous users of alcohol and cannabis differ from concurrent users in risk of cannabis-related problems after accounting for marijuana frequency and quantity? To our knowledge, this study represents the first analysis that investigated the association between SAM and cannabis-related problems in the adult population in the US.

## Methods

### Study population

The Washington liquor privatization panel surveys were conducted between September 2014 and April 2016 on selected respondents drawn from the first 4 waves of a series of representative cross-sectional surveys. Cross-sectional survey respondents were recruited using list-assisted dual-frame Random Digit Dial sampling of telephone numbers, including both landline household recruitment and cell phone recruitment (with > 40% from cell phones at each survey). Eligibility for panel follow-up was based on alcohol and cannabis use and included all respondents who were current (past-year) spirits drinkers or current cannabis users who also drank any alcoholic beverage. Detailed descriptions of the survey sample can be found elsewhere (Kerr et al. 2019). Protocols were approved by the Public Health Institute Institutional Review Board (#I13-010). In this study, we restricted the analytic sample to panel survey participants who reported drinking any kind of alcoholic beverage and using cannabis in the past 6 months, for whom the co-use status of alcohol and cannabis was assessed and the Cannabis Use Disorder Identification Test (CUDIT) was administered. (The CUDIT was not included in the baseline surveys, only in the Panel 1 to Panel 4 surveys.) A flowchart of the final panel sample can be found in Figure S1, Supplementary information.

### Measures

Past-six-month Cannabis Use Disorder Identification Test (CUDIT) is a 10-item score for assessing cannabis use in the panel data that ranges from 0 to 40 points. This screening instrument has been validated for identifying problematic cannabis users in previous studies (Adamson and Sellman 2003; Annaheim et al. 2008; Thake and Davis 2011). Past-six-month CUDIT problem subscale is the total score for seven CUDIT problem items (Cronbach’s alpha = 0.72) that reveal cannabis abuse or dependence symptoms (e.g., unable to stop using marijuana once started, needing to use marijuana in the morning, feeling of guilt or remorse after using marijuana, etc.). We excluded the three items from CUDIT that implied marijuana use frequency (i.e., How often have you used marijuana, hash or pot during the last six months? When you use marijuana or hashish, how long do you usually stay high? How often were you high for six or more hours?), which was included in the model as a predictor of interest. The CUDIT problem subscale ranges from 0 to 28. Full details of CUDIT subscale items can be found in Table S1 in the Supplementary information.

We determined the co-use status of alcohol and marijuana for the study participants based on the question, “When you used marijuana or marijuana products in the past 6 months, how often did you use alcohol at the same time? Was it [options] usually, sometimes or never?” Marijuana use frequency was derived from the question, “How often have you used marijuana, hash or pot during the last 6 months?” We recoded the variable into 3 categories: (a) daily/near daily use, (b) regular use (about once per week, once every 2–3 weeks, or once every month or two), and (c) infrequent use (less than every month or two). Based on these two questions and participants’ drinking status, we recoded co-use status of alcohol and marijuana use into simultaneous use (usually or sometimes used marijuana and alcohol at the same time) and concurrent use (used alcohol and marijuana in the past 6 months, but never use them at the same time).

In addition to this categorical measure of marijuana use frequency, we also included grams per day of marijuana use estimated from purchases (Kerr and Ye 2022) as a continuous measure of marijuana quantity, to adjust for the confounding effects of marijuana use more comprehensively. The alcohol volume was calculated from beverage-specific assessment as a total number of standard drinks reported in the past 6 months, after adjusting for drink and brand characteristics relevant to the size and ABV% (alcohol by volume percentage) of beer, wine, and spirits typically consumed (Kerr et al. 2005). One standard drink (14 g) was defined as a 12-ounce bottle/can of beer, a 5-ounce glass of wine, or a 1.5-ounce shot of liquor. We used a natural log transformation of marijuana daily quantity and beverage-specific alcohol volume because of their skewed distributions.

Other covariates included sex (female vs. male), race/ethnicity (Black, Hispanic, Others/Missing vs. White), age group (30–49, 50+, vs. 18–29), age of onset ( < = 17, 18–25, vs. >=26), annual household income (< 50k, 50-80k, vs. >80k), education (some college or more vs. high school or less), marital status (married vs. unmarried), employment status (employed vs. unemployed), having medical recommendation from a health care professional for marijuana or cannabis (yes vs. no), and time to nearest marijuana outlet (minutes).

### Statistical analyses

We used generalized estimating equations (GEE) with Poisson-distributed outcome (hereafter, Poisson GEE) to analyze the association between SAM (compared to concurrent use) and CUDIT problem subscale. GEE provides robust inference on parameters of interest and can account for within-subject correlation using sandwich-type variance estimates (Liang and Zeger 1986; Zhang et al. 2012). We specified exchangeable within-subject correlation and applied Huber-White sandwich estimator of variance, which can produce valid standard errors even in the case of correlation misspecification. In addition, GEE is very flexible with unbalanced data, i.e., when there are more observations for some participants than others, such as in the Washington panel data utilized here (Figure S1, Supplementary information).

Covariates included in the Poisson GEE models were selected *a priori* based on previous literature (Chen et al. 2022; Fischer et al. 2017; Robinson et al. 2022; Winters and Lee 2008). We adjusted for two different measures of marijuana use while analyzing the association between SAM and the CUDIT problem subscale: (1) marijuana use frequency (infrequent use, regular use, or daily/near daily use) and (2) marijuana daily quantity consumed estimated from purchases (Kerr and Ye 2022). The two measures represent two dimensions of marijuana use, which can account for the confounding effects more comprehensively (Callaghan et al. 2020). All models were adjusted for sex, race/ethnicity, education, age of marijuana use onset, age group, marital status, employment status, family income, panel survey cycle, medical marijuana recommendation, driving time to nearest marijuana outlet (minutes), and accounted for differential probability of selection and response rates through survey weights. Besides, in Model 1, we additionally adjusted for marijuana use frequency; in Model 2, we replaced marijuana use frequency with marijuana daily quantity consumed; in Model 3, we additionally adjusted for both measures of marijuana use; in Model 4, we additionally adjusted for both measures of marijuana use and alcohol volume. Additionally, we analyzed the association between SAM (compared to concurrent use) and the CUDIT total score. Because marijuana use frequency is already included in the CUDIT total, we compared models with and without adjusting for marijuana use frequency. We performed statistical analyses using R 4.1.3 (R Core Team, 2022) and Stata V.17, StataCorp, College Station, TX, USA (StataCorp 2021).

## Results

After excluding missing values (which accounted for 6% of the data) of the study sample, 257 participants remained in the final sample. Each participant had 1–4 observations throughout the four sets of panel surveys: 137 participants had only one observation, 73 participants had two observations, 35 participants had three observations, and 12 participants had four observations, resulting in 436 observations in total in the longitudinal data sample. Table 1 shows the weighted prevalence (for categorical variables) or weighted mean (SD) (for continuous variables) of the participants’ demographic characteristics in each panel survey. Chi-square tests (for categorical variables) or one-way ANOVA tests (for continuous variables) were performed to test for differences across survey waves. We observed a significant decreasing trend in average time to nearest marijuana outlet because legal recreational cannabis dispensaries were gradually introduced in Washington state from Sep 2014 to April 2016.

**Table 1:** Demographic Characteristics of Study Participants (weighted %, mean, and SD)

	Panel 1: Sept.-Nov. 2014 (*n *= 60)	Panel 2: March-June 2015 (*n *= 89)	Panel 3: August-Oct. 2015 (*n *= 130)	Panel 4: March-April 2016 (*n* = 157)	*p*-value
Sex (%)					0.99
Female	44%	42%	44%	44%	
Male	56%	58%	56%	56%	
Race/ethnicity (%)					0.32
White	78%	76%	79%	77%	
Black	10%	13%	6%	4%	
Hispanic	3%	3%	5%	11%	
Others/Missing	10%	8%	10%	9%	
Age group (%)					0.38
18-29	32%	42%	40%	37%	
30-49	48%	32%	28%	37%	
50+	19%	26%	33%	27%	
Age of onset (%)					0.71
<=17	65%	60%	52%	56%	
18-25	28%	32%	39%	33%	
>=26	6%	8%	9%	11%	
Household income (%)					0.06
<50k	68%	73%	60%	60%	
50-80k	26%	12%	15%	18%	
>80k	6%	15%	25%	22%	
Education (%)					0.29
High school or less	36%	34%	22%	31%	
Some college or more	64%	66%	78%	69%	
Employment status (%)					0.44
Unemployed	32%	31%	40%	31%	
Employed (full- or part-time)	68%	69%	60%	69%	
Marital status (%)					0.53
Married	41%	44%	45%	52%	
Unmarried	59%	56%	55%	48%	
Have medical cannabis recommendation (%)					0.18
Yes	40%	32%	24%	23%	
No	60%	68%	76%	77%	
Co-use of marijuana and alcohol (%)					0.09
Concurrent user	46%	38%	52%	57%	
Simultaneous co-use	54%	62%	48%	43%	
Marijuana use (%)					0.33
Infrequent	24%	17%	15%	22%	
Regular use	40%	30%	45%	37%	
Daily/near daily use	36%	53%	40%	41%	
Time to nearest marijuana outlet (minutes) (mean (SD))	20.1 (20.8)	11.5 (11.9)	9.0 (12.2)	8.2 (17.0)	0.003
Marijuana daily quantity (gram) (mean (SD))	0.3 (0.6)	0.4 (0.6)	0.2 (0.4)	0.3 (0.4)	0.35
Alcohol daily volume (mean (SD))	1.1 (1.4)	1.2 (1.6)	1.2 (1.4)	0.9 (1.0)	0.43

Results of GEE Poisson regressions with CUDIT problem subscale are presented in Table 2. We found that compared to concurrent use, SAM was significantly positively associated with CUDIT problem subscale in all models, even after adjusting for marijuana use frequency and daily quantity, and alcohol volume. Daily/near daily use of marijuana was strongly significantly associated with CUDIT problem subscale compared with infrequent use or regular use (Model 1), even after adjusting for marijuana daily quantity and alcohol volume (Model 3 and Model 4). Marijuana daily quantity was positively significantly associated with CUDIT problem subscale (Model 2), however, the effect was attenuated towards the null and became insignificant after additionally adjusting for categorical marijuana use frequency (Model 3 and Model 4).

**Table 2:** Adjusted Incidence Rate Ratios (IRR, 95% CI) between predictors and CUDIT problem subscale (Range: 0–28) from Poisson GEE models

	Model 1	Model 2	Model 3	Model 4
Co-use of alcohol and marijuana				
SAM vs. Concurrent	1.59, (1.18, 2.13)**	1.69, (1.22, 2.35)**	1.62, (1.21, 2.18)**	1.68, (1.25, 2.27)***
Marijuana use frequency				
Regular vs. Infrequent	1.89, (0.97, 3.71)	-	1.68, (0.84, 3.38)	1.67, (0.83, 3.36)
Daily/near daily vs. Infrequent	6.88, (3.76, 12.58)***	-	5.2, (2.79, 9.69)***	5.1, (2.71, 9.57)***
Daily/near daily vs. Regular	3.63, (2.22, 5.93)***	-	3.09, (1.94, 4.91)***	3.05, (1.91, 4.85)***
Log marijuana daily quantity	-	1.33, (1.18, 1.48)***	1.1, (1, 1.21)	1.1, (0.99, 1.21)
Log alcohol volume	-	-	-	0.98, (0.87, 1.09)
Time to nearest marijuana outlet	0.99, (0.98, 1.01)	1, (0.98, 1.01)	1, (0.98, 1.01)	1, (0.98, 1.01)
Medical marijuana recommendation (ref: no)				
Have medical recommendation	0.8, (0.51, 1.25)	0.91, (0.59, 1.41)	0.81, (0.53, 1.25)	0.8, (0.52, 1.24)
Sex (ref: male)				
Female	0.86, (0.53, 1.38)	0.87, (0.54, 1.4)	0.9, (0.55, 1.46)	0.89, (0.55, 1.45)
Race/ethnicity (ref: White)				
Black	1.09, (0.33, 3.64)	1.01, (0.28, 3.67)	1.02, (0.3, 3.44)	1.05, (0.31, 3.53)
Hispanic	1.05, (0.31, 3.6)	1.16, (0.43, 3.15)	1.05, (0.3, 3.67)	1.07, (0.31, 3.7)
Others/missing	1.59, (0.86, 2.94)	1.82, (0.92, 3.59)	1.67, (0.92, 3.03)	1.65, (0.9, 3.03)
Education (ref: less than high school)				
Some college or more	0.98, (0.6, 1.61)	0.96, (0.59, 1.56)	1.01, (0.61, 1.67)	1.02, (0.62, 1.68)
Age group (ref: 18–29)				
30–49	0.55, (0.31, 0.99)*	0.47, (0.27, 0.8)**	0.56, (0.32, 0.98)*	0.55, (0.31, 0.98)*
>=50	0.55, (0.28, 1.07)	0.55, (0.29, 1.07)	0.57, (0.3, 1.08)	0.56, (0.3, 1.06)
Onset age of marijuana use (ref: >=26)				
<=17	0.83, (0.35, 1.96)	0.75, (0.32, 1.76)	0.8, (0.34, 1.87)	0.79, (0.34, 1.83)
18–25	0.66, (0.26, 1.66)	0.53, (0.21, 1.31)	0.64, (0.26, 1.59)	0.64, (0.27, 1.5)
Family annual income (ref: >80,000)				
<50,000	0.75, (0.43, 1.31)	0.85, (0.5, 1.43)	0.71, (0.41, 1.21)	0.71, (0.41, 1.22)
$50,000–80,000	0.74, (0.37, 1.46)	0.95, (0.48, 1.89)	0.72, (0.37, 1.42)	0.73, (0.37, 1.43)
Marital status (ref: unmarried)				
Married	0.6, (0.39, 0.92)*	0.67, (0.42, 1.07)	0.6, (0.39, 0.93)*	0.6, (0.38, 0.93)*
Employment status (ref: unemployed/retired/etc.)				
Full-time or part-time	1.04, (0.59, 1.85)	0.92, (0.53, 1.61)	1.02, (0.59, 1.77)	1.03, (0.58, 1.82)
Survey cycle [ref: panel 1 (Sept.-Nov. 2014)]				
Panel 2 (March-June 2015)	0.78, (0.49, 1.23)	0.94, (0.54, 1.62)	0.79, (0.5, 1.25)	0.78, (0.5, 1.24)
Panel 3 (August-Oct. 2015)	1.13, (0.78, 1.66)	1.46, (0.89, 2.38)	1.16, (0.78, 1.73)	1.17, (0.78, 1.74)
Panel 4 (March-April 2016)	0.81, (0.56, 1.19)	1, (0.62, 1.63)	0.82, (0.55, 1.22)	0.83, (0.56, 1.23)

All models were adjusted for sex, race/ethnicity, education, age of marijuana use onset, age group, marital status, employment status, family income, panel survey cycle, medical marijuana recommendation, driving time to nearest marijuana outlet (minutes), and accounted for differential probability of selection and response rates through survey weights. Besides, in Model 1, we additionally adjusted for marijuana use frequency; in Model 2, we replaced marijuana use frequency with natural log transformed marijuana daily quantity consumed; in Model 3, we additionally adjusted for both measures of marijuana use; in Model 4, we additionally adjusted for both measures of marijuana use and natural log transformed alcohol volume

*p < 0.05

**p < 0.01

***p < 0.001

Results of GEE Poisson regressions with CUDIT total score are shown in Table 3. The positive associations between SAM (compared to concurrent use) and the CUDIT total were attenuated compared to those with CUDIT problem subscale yet still remained significant in all models (Models 5–7), even after additionally adjusting for marijuana use frequency (Model 8), which was accounted for the CUDIT total score. Marijuana daily quantity was also significantly associated with CUDIT total even after adjusting for marijuana use frequency (Model 7 and Model 8). No multicollinearity was identified in these models.

**Table 3:** Adjusted Incidence Rate Ratios (IRR, 95% CI) between predictors and CUDIT total score (Range: 0–40) from Poisson GEE

	Model 5	Model 6	Model 7	Model 8
Co-use of alcohol and marijuana				
SAM vs. Concurrent	1.22, (1.01, 1.46)*	1.22, (1.02, 1.47)*	1.22, (1.05, 1.42)*	1.17, (1.02, 1.34)*
Marijuana use frequency				
Regular vs. Infrequent	-	-	-	1.66, (1.27, 2.18)***
Daily/near daily vs. Infrequent	-	-	-	3.3, (2.44, 4.46)***
Daily/near daily vs. Regular	-	-	-	1.98, (1.65, 2.39)***
Log marijuana daily quantity	-	-	1.25, (1.18, 1.33)***	1.1, (1.04, 1.16)***
Log alcohol volume	-	0.99, (0.94, 1.04)	0.99, (0.95, 1.03)	1, (0.97, 1.04)
Time to nearest marijuana outlet	1, (0.99, 1.01)	1, (0.99, 1.01)	1, (0.997, 1.004)	0.998, (0.995, 1.002)
Medical marijuana recommendation (ref: no)				
Have medical recommendation	1.18, (0.95, 1.48)	1.18, (0.94, 1.47)	1.12, (0.95, 1.32)	1.01, (0.86, 1.19)
Sex (ref: male)				
Female	0.7, (0.56, 0.88)**	0.7, (0.56, 0.88)**	0.84, (0.69, 1.02)	0.87, (0.73, 1.05)
Race/ethnicity (ref: White)				
Black	1.16, (0.74, 1.83)	1.17, (0.74, 1.84)	0.93, (0.66, 1.32)	0.94, (0.7, 1.27)
Hispanic	1.17, (0.76, 1.78)	1.17, (0.77, 1.79)	1.2, (0.85, 1.7)	1.13, (0.75, 1.69)
Others/missing	1.1, (0.65, 1.87)	1.1, (0.65, 1.86)	1.31, (0.88, 1.94)	1.23, (0.9, 1.69)
Education (ref: less than high school)				
Some college or more	0.84, (0.67, 1.06)	0.84, (0.67, 1.06)	0.95, (0.79, 1.14)	0.94, (0.79, 1.11)
Age group (ref: 18–29)				
30–49	0.64, (0.48, 0.84)**	0.64, (0.48, 0.84)**	0.7, (0.56, 0.87)**	0.79, (0.64, 0.97)*
>=50	0.71, (0.52, 0.98)*	0.72, (0.52, 0.99)*	0.82, (0.64, 1.07)	0.83, (0.65, 1.07)
Onset age of marijuana use (ref: >=26)				
<=17	1.05, (0.72, 1.53)	1.05, (0.72, 1.53)	0.91, (0.65, 1.27)	0.96, (0.69, 1.33)
18–25	0.85, (0.59, 1.23)	0.85, (0.6, 1.21)	0.77, (0.55, 1.06)	0.88, (0.64, 1.22)
Family annual income (ref: >80,000)				
<50,000	1.27, (0.94, 1.73)	1.27, (0.94, 1.72)	1.03, (0.8, 1.32)	0.89, (0.71, 1.11)
$50,000–80,000	1.1, (0.76, 1.58)	1.1, (0.76, 1.57)	1.03, (0.77, 1.37)	0.87, (0.67, 1.12)
Marital status (ref: unmarried)				
Married	0.94, (0.74, 1.2)	0.94, (0.74, 1.2)	0.9, (0.75, 1.08)	0.85, (0.72, 1.01)
Employment status (ref: unemployed/retired/etc.)				
Full-time or part-time	0.92, (0.7, 1.2)	0.92, (0.7, 1.21)	0.94, (0.76, 1.16)	0.98, (0.79, 1.21)
Survey cycle [ref: panel 1 (Sept.-Nov. 2014)]				
Panel 2 (March-June 2015)	1.11, (0.85, 1.44)	1.11, (0.85, 1.44)	1.12, (0.87, 1.44)	1.01, (0.82, 1.24)
Panel 3 (August-Oct. 2015)	1.2, (0.93, 1.54)	1.2, (0.93, 1.54)	1.23, (0.97, 1.56)	1.08, (0.89, 1.31)
Panel 4 (March-April 2016)	1.12, (0.88, 1.42)	1.12, (0.88, 1.42)	1.08, (0.87, 1.33)	0.96, (0.81, 1.13)

All models were adjusted for sex, race/ethnicity, education, age of marijuana use onset, age group, marital status, employment status, family income, panel survey cycle, medical marijuana recommendation, driving time to nearest marijuana outlet (minutes), and accounted for differential probability of selection and response rates through survey weights. Besides, in Model 6, we additionally adjusted for natural log transformed alcohol volume; in Model 7, we additionally adjusted for natural log transformed marijuana daily quantity and alcohol volume consumed; in Model 8, we additionally adjusted for marijuana use frequency, natural log transformed marijuana daily quantity and alcohol volume

*p < 0.05

**p < 0.01

***p < 0.001

Sensitivity analyses adjusting for both linear and quadratic terms of marijuana daily quantity estimated from purchases led to similar associations between SAM (compared to concurrent use) and CUDIT problem subscale (Model S1 and Model S2 in Table S2, Supplementary information). Marijuana use frequency remained a strong predictor of CUDIT problem subscale in the model, although the quadratic term of marijuana daily quantity was not significantly associated with CUDIT problem subscale. Similar to Callaghan et al. (2020), we also explored the potential interactions between marijuana use frequency and daily quantity, but did not observe any significant findings. Sensitivity analyses using GEE with negative binomial-distributed outcome generated similar results to those with Poisson-distributed outcome (Table S3, Table S4, and Table S5, Supplementary information).

## Discussion

This study makes a unique contribution to the literature by establishing that simultaneous users of alcohol and marijuana were significantly more likely than concurrent users to have cannabis-related problems as measured by the CUDIT problem subscale in a representative longitudinal data sample of adult cannabis and alcohol users in Washington State following the RCL, even after statistically adjusting for marijuana use frequencies and quantities. This finding suggests that simultaneous use of alcohol and marijuana has a synergistic effect that contributes not only to alcohol-related problems, as showed by previous studies (McCabe et al. 2006; Midanik et al. 2007; Subbaraman and Kerr 2015, 2018), but also to cannabis-related problems in the state-representative population of adult drinkers and marijuana users. Because SAM is a prevalent co-use pattern in young people, most of the previous studies on SAM focused on adolescents or young adults (Martin et al. 1996; Terry-McElrath et al. 2013; Terry-McElrath et al. 2018; Patrick et al. 2018; Patrick et al. 2019), while this study included adults aged 21 years and older, who were the target of RCL. In addition, we used longitudinal data with multiple observations per individual and accounted for within-person correlation through the statistical approach, which can better establish the cause-and-effect relationship between SAM and cannabis-related problems compared to cross-sectional studies. To our knowledge, this was the first study that demonstrated the linkage between SAM and cannabis-related problems in an adult population.

This study has important implications for interventions. With the legalization of recreational marijuana use in people aged 21 years and older, SAM became a more prevalent co-use pattern than before in this population (Gonçalves et al. 2022), which suggests the complementarity hypothesis, i.e., alcohol and marijuana are used to enhance the effects of each other (Subbaraman 2016; Gunn et al. 2022). As SAM is associated with increased risks of not only alcohol-related outcomes but also cannabis-related problems, it is important to target simultaneous users of alcohol and marijuana for future interventions, especially among those aged 21 years and older. Specifically, current policies should be sustained to keep alcohol and cannabis products, sales and use contexts separate; and primary care clinicians should consider screening for substance co-use such as SAM in alcohol users and providing brief interventions and referral to appropriate treatments, especially in states that legalized cannabis for recreational use.

This study also has some limitations. First, our data were collected based on self-report, without validated biomedical measures or collateral informant reports. However, a comparison study based on a large-scale clinical trial suggests that such alternative measures do not greatly add to the accuracy of self-report measurements (Babor et al. 2000). Second, although we adjusted for survey weights in the statistical analyses, findings of this study were derived from the specific state of Washington, which may not be generalizable to other states with RCL in the US. Future work is needed to examine whether the findings can be replicated in other populations. Third, although we controlled for confounding factors as best as we could, we cannot rule out the possibility of residual confounding. For example, previous studies suggested that alcohol and marijuana use contexts (park, beach, car, or party) might also confound the relationship between SAM and cannabis-related problems (Terry-McElrath et al. 2013). Fourth, GEE uses a quasi-likelihood estimation without specifying the joint distribution of a participant’s observations (Liang and Zeger 1986); thus maximum likelihood estimation (MLE) tools for model selection are not suitable here. However, we fitted multiple models adjusting for different sets of covariates, which led to the robust finding that SAM was statistically significantly linked to higher cannabis-related problems compared to concurrent use. Thus, the lack of formal tests for model comparison should not have influenced our results.

## Conclusions

This study has highlighted the importance of SAM, in addition to cannabis use frequency for predicting cannabis-related problems as measured in the CUDIT problem subscale in adults. These results indicate a need for prevention and intervention efforts that target adult simultaneous users of alcohol and cannabis to reduce cannabis-related problems in addition to alcohol abuse/dependence, and sustain current policies designed to keep alcohol and cannabis products, sales and use contexts separate.

### Supplementary Information

**Supplementary Material 1.**

## Data Availability

The datasets used in this study are not publicly available because the project is ongoing and because the dataset includes potentially identifying information such as location-based measures. A public use version of the data will be made available at the end of the project in 2024. The data may also be made available by the Principal Investigator William C. Kerr for specific purposes with a data use agreement on reasonable request.

## Abbreviations

SAM: Simultaneous use of alcohol and marijuana
CUDIT: Cannabis use disorder identification test
