# Supplementary material for: Associations between simultaneous use of alcohol and cannabis and cannabis-related problems in 2014–2016: evidence from the Washington panel survey
Source: J Cannabis Res. 2024 Feb 24;6:8. doi: 10.1186/s42238-024-00217-z (PMC10893643; doi:10.1186/s42238-024-00217-z)
Supplement: Supplementary file 1 — Supplementary Material 1. [file 42238_2024_217_MOESM1_ESM.docx]

**Supplementary information for**

**Associations between Simultaneous Use of Alcohol and Cannabis and Cannabis-related Problems in 2014-2016: Evidence from the Washington Panel Survey**

Yachen Zhu^1^*, Yu Ye^1^, Thomas K. Greenfield^1^, William C. Kerr^1^

^1^Alcohol Research Group, Public Health Institute, Emeryville, CA 94608, USA

*Correspondence: yzhu@arg.org

List of Figures and Tables:

**Figure S1.** Diagram of the Washington panel surveys.

**Table S1.** The Cannabis Use Disorders Identification Test (CUDIT) Problem Subscale (Range: 0-28).

**Table S2.** Adjusted Incidence Rate Ratios (IRR, 95% CI) between Predictors and CUDIT Problem Subscale (Range: 0-28) from Poisson GEE.

**Table S3.** Adjusted Incidence Rate Ratios (IRR, 95% CI) between Predictors and CUDIT Problem Subscale (Range: 0-28) from Negative Binomial GEE.

**Table S4.** Adjusted Incidence Rate Ratios (IRR, 95% CI) between Predictors and CUDIT Problem Subscale (Range: 0-28) from Negative Binomial GEE.

**Table S5.** Adjusted Incidence Rate Ratios (IRR, 95% CI) between Predictors and CUDIT Total Score (Range: 0-40) from Negative Binomial GEE.


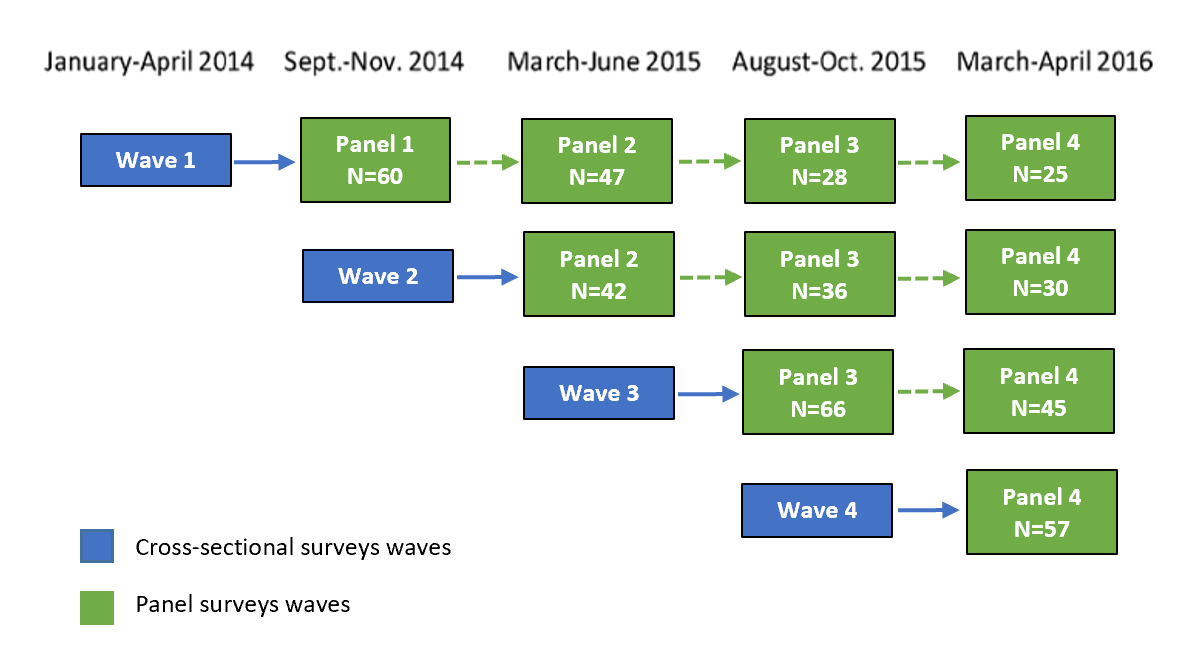


**Figure S1.** Diagram of the Washington panel surveys (modified with permission from Kerr et al., 2019).

**Table S1.** The Cannabis Use Disorders Identification Test (CUDIT) Problem Subscale (Range: 0-28).

| Have you used any marijuana, hash or pot during the last six months? If YES, please answer the following questions about your cannabis use |  | Answers |
| --- | --- | --- |
| 1. How often did you find that you were not able to stop using marijuana once you had started? |  | never (0), less than monthly (1), monthly (2), weekly (3), daily or almost daily (4) |
| 1. How often did you fail to do what was normally expected from you because of using marijuana? |  | never (0), less than monthly (1), monthly (2), weekly (3), daily or almost daily (4) |
| 1. How often did you need to use marijuana in the morning to get yourself going after a heavy session of using marijuana? |  | never (0), less than monthly (1), monthly (2), weekly (3), daily or almost daily (4) |
| 1. How often did you have a feeling of guilt or remorse after using marijuana? |  | never (0), less than monthly (1), monthly (2), weekly (3), daily or almost daily (4) |
| 1. How often have you had a problem with your memory or concentration after using marijuana? |  | never (0), less than monthly (1), monthly (2), weekly (3), daily or almost daily (4) |
| 1. Have you or someone else been injured as a result of your use of marijuana? |  | no (0), yes (4) |
| 1. Has a relative, friend or doctor or other health worker been concerned about your use of marijuana or suggested you cut down? |  | no (0), yes (4) |

**Table S2.** Adjusted Incidence Rate Ratios (IRR, 95% CI) between Predictors and CUDIT Problem Subscale (Range: 0-28) from Poisson GEE.

|  | Model S1 | Model S2 |
| --- | --- | --- |
| Co-use of alcohol and marijuana |  |  |
| SAM vs. Concurrent use | **1.67, (1.19, 2.33)**** | **1.62, (1.2, 2.2)**** |
| Marijuana use frequency |  |  |
| Regular vs. Infrequent | - | 1.9, (0.96, 3.74) |
| Daily/near daily vs. Infrequent | - | **6.48, (3.46, 12.13)***** |
| Daily/near daily vs. Regular | - | **3.41, (2.06, 5.67)***** |
| Marijuana daily quantity | **1.99, (1.09, 3.61)*** | 1.22, (0.79, 1.89) |
| Marijuana daily quantity squared | 0.88, (0.77, 1.02) | 0.97, (0.9, 1.06) |
| Time to nearest marijuana outlet | 1, (0.98, 1.01) | 1, (0.98, 1.01) |
| Medical marijuana recommendation (ref: no) |  |  |
| Have medical recommendation | 0.93, (0.58, 1.51) | 0.8, (0.52, 1.24) |
| Sex (ref: male) |  |  |
| Female | 0.76, (0.47, 1.24) | 0.87, (0.54, 1.42) |
| Race/ethnicity (ref: White) |  |  |
| Black | 1.41, (0.42, 4.67) | 1.08, (0.32, 3.6) |
| Hispanic | 1.21, (0.51, 2.86) | 1.08, (0.31, 3.73) |
| Others/missing | 1.61, (0.76, 3.38) | 1.62, (0.89, 2.95) |
| Education (ref: less than high school) |  |  |
| Some college or more | 0.85, (0.53, 1.36) | 1, (0.6, 1.64) |
| Age group (ref: 18-29) |  |  |
| 30-49 | **0.4, (0.23, 0.7)**** | **0.56, (0.32, 0.98)*** |
| >=50 | **0.49, (0.25, 0.96)*** | 0.55, (0.29, 1.06) |
| Onset age of marijuana use (ref: >=26) |  |  |
| <=17 | 0.8, (0.32, 1.99) | 0.82, (0.34, 1.93) |
| 18-25 | 0.56, (0.21, 1.47) | 0.65, (0.26, 1.65) |
| Family annual income (ref: >80,000) |  |  |
| <50,000 | 0.91, (0.52, 1.6) | 0.72, (0.4, 1.3) |
| $50,000-80,000 | 0.92, (0.44, 1.91) | 0.72, (0.36, 1.43) |
| Marital status (ref: unmarried) |  |  |
| Married | 0.69, (0.42, 1.13) | **0.6, (0.39, 0.94)*** |
| Employment status (ref: unemployed/retired/etc.) |  |  |
| Full-time or part-time | 0.91, (0.5, 1.65) | 1.02, (0.58, 1.8) |
| Survey cycle [ref: panel 1 (Sept.-Nov. 2014)] |  |  |
| Panel 2 (March-June 2015) | 1.02, (0.55, 1.89) | 0.81, (0.5, 1.3) |
| Panel 3 (August-Oct. 2015) | 1.49, (0.84, 2.63) | 1.18, (0.77, 1.8) |
| Panel 4 (March-April 2016) | 1.1, (0.65, 1.86) | 0.85, (0.56, 1.27) |

Model S1 and Model S2 adjusted for marijuana daily quantity and marijuana daily quantity squared instead of natural log transformation of marijuana daily quantity.

*p<0.05

**p<0.01

***p<0.001

**Table S3.** Adjusted Incidence Rate Ratios (IRR, 95% CI) between Predictors and CUDIT Problem Subscale (Range: 0-28) from Negative Binomial GEE.

|  | Model S3 | Model S4 | Model S5 | Model S6 |
| --- | --- | --- | --- | --- |
| Co-use of alcohol and marijuana |  |  |  |  |
| SAM vs. Concurrent use | **1.65, (1.19, 2.30)**** | **1.65, (1.15, 2.38)**** | **1.67, (1.19, 2.34)**** | **1.75, (1.24, 2.47)**** |
| Marijuana use frequency |  |  |  |  |
| Regular vs. Infrequent | 1.99, (0.96, 4.09) | - | 1.74, (0.83, 3.65) | 1.73, (0.83, 3.64) |
| Daily/near daily vs. Infrequent | **8.29, (4.37, 15.71)***** | - | **6.08, (3.15, 11.78)***** | **5.98, (3.08, 11.60)***** |
| Daily/near daily vs. Regular | **4.18, (2.67, 6.52)***** | - | **3.49, (2.28, 5.35)***** | **3.45, (2.25, 5.29)***** |
| Log marijuana daily quantity | - | **1.38, (1.24, 1.54)***** | 1.11, (0.99, 1.24) | 1.10, (0.99, 1.24) |
| Log alcohol volume | - | - | - | 0.97, (0.87, 1.08) |
| Time to nearest marijuana outlet | 1.00, (0.99, 1.01) | 1.00, (0.99, 1.01) | 1.00, (0.99, 1.01) | 1.00, (0.99, 1.01) |
| Medical marijuana recommendation (ref: no) |  |  |  |  |
| Have medical recommendation | 0.68, (0.43, 1.06) | 0.91, (0.59, 1.42) | 0.68, (0.44, 1.05) | 0.68, (0.44, 1.05) |
| Sex (ref: male) |  |  |  |  |
| Female | 1.05, (0.64, 1.70) | 1.01, (0.62, 1.65) | 1.09, (0.66, 1.79) | 1.08, (0.65, 1.79) |
| Race/ethnicity (ref: White) |  |  |  |  |
| Black | 1.04, (0.41, 2.67) | 0.92, (0.32, 2.64) | 0.98, (0.39, 2.52) | 0.99, (0.39, 2.54) |
| Hispanic | 0.83, (0.22, 3.18) | 0.88, (0.30, 2.63) | 0.87, (0.23, 3.26) | 0.88, (0.23, 3.27) |
| Others/missing | 1.20, (0.53, 2.73) | 1.33, (0.60, 2.96) | 1.27, (0.55, 2.93) | 1.27, (0.55, 2.94) |
| Education (ref: less than high school) |  |  |  |  |
| Some college or more | 0.89, (0.52, 1.52) | 0.89, (0.54, 1.49) | 0.89, (0.52, 1.53) | 0.90, (0.53, 1.55) |
| Age group (ref: 18-29) |  |  |  |  |
| 30-49 | 0.68, 0.37, 1.25) | **0.57, (0.33, 0.99)** | 0.69, (0.38, 1.24) | 0.68, (0.38, 1.23) |
| >=50 | 0.62, (0.33, 1.18) | 0.67, (0.36, 1.24) | 0.65, (0.35, 1.23) | 0.64, (0.34, 1.19) |
| Onset age of marijuana use (ref: >=26) |  |  |  |  |
| <=17 | 1.00, (0.45, 2.23) | 0.93, (0.42, 2.06) | 0.98, (0.45, 2.15) | 1.00, (0.46, 2.18) |
| 18-25 | 0.77, (0.33, 1.80) | 0.67, (0.28, 1.58) | 0.76, (0.33, 1.76) | 0.79, (0.35, 1.77) |
| Family annual income (ref: >80,000) |  |  |  |  |
| <50,000 | 0.70, (0.35, 1.42) | 0.86, (0.46, 1.60) | 0.68, (0.34, 1.36) | 0.67, (0.33, 1.35) |
| $50,000-80,000 | 0.72, (0.34, 1.49) | 1.05, (0.53, 2.08) | 0.73, (0.35, 1.50) | 0.73, (0.35, 1.50) |
| Marital status (ref: unmarried) |  |  |  |  |
| Married | 0.64, (0.40, 1.01) | 0.70, (0.44, 1.10) | 0.64, (0.40, 1.00) | 0.64, (0.40, 1.01) |
| Employment status (ref: unemployed/retired/etc.) |  |  |  |  |
| Full-time or part-time | 1.02, (0.57, 1.79) | 0.99, (0.58, 1.69) | 1.02, (0.59, 1.78) | 1.02, (0.58, 1.78) |
| Survey cycle [ref: panel 1 (Sept.-Nov. 2014)] |  |  |  |  |
| Panel 2 (March-June 2015) | 0.73, (0.46, 1.14) | 0.96, (0.60, 1.54) | 0.75, (0.49, 1.15) | 0.75, (0.49, 1.16) |
| Panel 3 (August-Oct. 2015) | 1.08, (0.71, 1.65) | 1.37, (0.88, 2.13) | 1.11, (0.73, 1.70) | 1.12, (0.74, 1.70) |
| Panel 4 (March-April 2016) | 0.82, (0.54, 1.24) | 0.97, (0.62, 1.66) | 0.82, (0.54, 1.25) | 0.82, (0.54, 1.25) |

Note: All models were adjusted for sex, race/ethnicity, education, age of marijuana use onset, age group, marital status, employment status, family income, panel survey cycle, medical marijuana recommendation, driving time to nearest marijuana outlet (minutes), and accounted for differential probability of selection and response rates through survey weights. Besides, in Model S3, we additionally adjusted for marijuana use frequency; in Model S4, we replaced marijuana use frequency with natural log transformed marijuana daily quantity consumed; in Model S5, we additionally adjusted for both measures of marijuana use; in Model S6, we additionally adjusted for both measures of marijuana use and natural log transformed alcohol volume.

*p<0.05

**p<0.01

***p<0.001

**Table S4.** Adjusted Incidence Rate Ratios (IRR, 95% CI) between Predictors and CUDIT Problem Subscale (Range: 0-28) from Negative Binomial GEE.

|  | Model S7 | Model S8 |
| --- | --- | --- |
| Co-use of alcohol and marijuana |  |  |
| SAM vs. Concurrent use | **1.63, (1.12, 2.37)*** | **1.51, (1.08, 2.13)*** |
| Marijuana use frequency | - |  |
| Regular vs. Infrequent |  | **2.21, (1.09, 4.48)*** |
| Daily/near daily vs. Infrequent |  | **8.72, (4.53, 16.79)***** |
| Daily/near daily vs. Regular |  | **3.95, (2.46, 6.35)***** |
| Marijuana daily quantity | 1.50, (0.69, 3.28) | 1.04, (0.60, 1.82) |
| Marijuana daily quantity squared | 0.98, (0.78, 1.22) | 1.01, (0.90, 1.13) |
| Time to nearest marijuana outlet | 0.99, (0.98, 1.01) | 1.00, (0.98, 1.01) |
| Medical marijuana recommendation (ref: no) |  |  |
| Have medical recommendation | 1.13, (0.69, 1.88) | 0.75, (0.48, 1.19) |
| Sex (ref: male) |  |  |
| Female | 0.90, (0.53, 1.53) | 1.14, (0.69, 1.89) |
| Race/ethnicity (ref: White) |  |  |
| Black | 1.02, (0.37, 2.80) | 0.93, (0.38, 2.25) |
| Hispanic | 0.76, (0.28, 2.06) | 0.86, (0.24, 3.13) |
| Others/missing | 1.00, (0.42, 2.38) | 1.03, (0.45, 2.34) |
| Education (ref: less than high school) |  |  |
| Some college or more | 0.73, (0.45, 1.20) | 0.79, (0.46, 1.36) |
| Age group (ref: 18-29) |  |  |
| 30-49 | 0.47, (0.27, 0.81) | 0.64, (0.35, 1.17) |
| >=50 | 0.55, (0.28, 1.07) | 0.64, (0.32, 1.27) |
| Onset age of marijuana use (ref: >=26) |  |  |
| <=17 | 0.97, (0.39, 2.44) | 1.12, (0.47, 2.63) |
| 18-25 | 0.62, (0.23, 1.67) | 0.79, (0.32, 1.93) |
| Family annual income (ref: >80,000) |  |  |
| <50,000 | 1.17, (0.61, 2.25) | 0.82, (0.39, 1.71) |
| $50,000-80,000 | 1.33, (0.66, 2.69) | 0.97, (0.46, 2.04) |
| Marital status (ref: unmarried) |  |  |
| Married | 0.72, (0.45, 1.16) | 0.65, (0.41, 1.05) |
| Employment status (ref: unemployed/retired/etc.) |  |  |
| Full-time or part-time | 0.96, (0.53, 1.72) | 0.99, (0.55, 1.80) |
| Survey cycle [ref: panel 1 (Sept.-Nov. 2014)] |  |  |
| Panel 2 (March-June 2015) | 1.11, (0.53, 2.32) | 1.73, (0.44, 1.22) |
| Panel 3 (August-Oct. 2015) | 1.39, (0.71, 2.73) | 1.02, (0.63, 1.65) |
| Panel 4 (March-April 2016) | 1.23, (0.65, 2.30) | 0.85, (0.54, 1.35) |

Model S7 and Model S8 adjusted for marijuana daily quantity and marijuana daily quantity squared instead of natural log transformation of marijuana daily quantity.

*p<0.05

**p<0.01

***p<0.001

**Table S5.** Adjusted Incidence Rate Ratios (IRR, 95% CI) between Predictors and CUDIT Total Score (Range: 0-40) from Negative Binomial GEE.

|  | Model S9 | Model S10 | Model S11 |
| --- | --- | --- | --- |
| Co-use of alcohol and marijuana |  |  |  |
| SAM vs. Concurrent use | **1.23, (1.06, 1.41)**** | **1.22, (1.06, 1.39)**** | **1.22, (1.06, 1.40)**** |
| Marijuana use frequency |  |  |  |
| Regular vs. Infrequent | **1.81, (1.41, 2.33)***** | **1.65, (1.28, 2.14)***** | **1.65, (1.28, 2.14)***** |
| Daily/near daily vs. Infrequent | **4.02, (3.05, 5.30)***** | **3.27, (2.43, 4.42)***** | **3.27, (2.43, 4.42)***** |
| Daily/near daily vs. Regular | **2.22, (1.84, 2.68)***** | **1.98, (1.65, 2.38)***** | **1.98, (1.65, 2.38)***** |
| Log marijuana daily quantity | - | **1.11, (1.05, 1.17)***** | **1.11, (1.05, 1.17)***** |
| Log alcohol volume | - | - | 1.00, (0.96, 1.04) |
| Time to nearest marijuana outlet | 1.00, (0.996, 1.004) | 1.00, (0.996, 1.003) | 1.00, (0.996, 1.003) |
| Medical marijuana recommendation (ref: no) |  |  |  |
| Have medical recommendation | 0.98, (0.84, 1.16) | 0.97, (0.83, 1.13) | 0.97, (0.83, 1.13) |
| Sex (ref: male) |  |  |  |
| Female | 0.84, (0.70, 1.02) | 0.90, (0.75, 1.08) | 0.90, (0.75, 1.08) |
| Race/ethnicity (ref: White) |  |  |  |
| Black | 0.98, (0.75, 1.29) | 0.98, (0.77, 1.25) | 0.98, (0.76, 1.25) |
| Hispanic | 1.03, (0.63, 1.70) | 1.08, (0.66, 1.77) | 1.08, (0.66, 1.77) |
| Others/missing | 0.96, (0.66, 1.39) | 1.03, (0.72, 1.48) | 1.03, (0.72, 1.48) |
| Education (ref: less than high school) |  |  |  |
| Some college or more | 0.90, (0.74, 1.09) | 0.90, (0.74, 1.09) | 0.90, (0.74, 1.08) |
| Age group (ref: 18-29) |  |  |  |
| 30-49 | 0.82, (0.65, 1.04) | 0.82, (0.66, 1.03) | 0.82, (0.66, 1.03) |
| >=50 | 0.81, (0.63, 1.03) | 0.85, (0.67, 1.08) | 0.85, (0.67, 1.08) |
| Onset age of marijuana use (ref: >=26) |  |  |  |
| <=17 | 1.08, (0.81, 1.45) | 1.02, (0.76, 1.35) | 1.02, (0.76, 1.35) |
| 18-25 | 1.01, (0.76, 1.33) | 0.96, (0.73, 1.27) | 0.96, (0.74, 1.26) |
| Family annual income (ref: >80,000) |  |  |  |
| <50,000 | 1.03, (0.79, 1.35) | 0.95, (0.74, 1.23) | 0.95, (0.74, 1.23) |
| $50,000-80,000 | 0.99, (0.74, 1.31) | 0.97, (0.74, 1.28) | 0.97, (0.74, 1.28) |
| Marital status (ref: unmarried) |  |  |  |
| Married | 0.92, (0.77, 1.11) | 0.91, (0.77, 1.07) | 0.91, (0.77, 1.07) |
| Employment status (ref: unemployed/retired/etc.) |  |  |  |
| Full-time or part-time | 0.98, (0.79, 1.22) | 0.98, (0.80, 1.19) | 0.98, (0.80, 1.19) |
| Survey cycle [ref: panel 1 (Sept.-Nov. 2014)] |  |  |  |
| Panel 2 (March-June 2015) | 1.00, (0.82, 1.22) | 1.01, (0.84, 1.22) | 1.01, (0.84, 1.22) |
| Panel 3 (August-Oct. 2015) | 1.08, (0.89, 1.29) | 1.09, (0.90, 1.31) | 1.09, (0.90, 1.31) |
| Panel 4 (March-April 2016) | 0.97, (0.82, 1.16) | 0.96, (0.81, 1.14) | 0.96, (0.81, 1.14) |

Note: Models S9-11 were adjusted for sex, race/ethnicity, education, age of marijuana use onset, age group, marital status, employment status, family income, panel survey cycle, medical marijuana recommendation, driving time to nearest marijuana outlet (minutes), marijuana use frequency, and accounted for differential probability of selection and response rates through survey weights. Besides, in Model S10, we additionally adjusted for natural log transformed marijuana daily quantity consumed; in Model S11, we additionally adjusted for natural log transformed marijuana daily quantity and alcohol volume. The model replaced marijuana use frequency with natural log transformed marijuana daily quantity failed with the error “estimates diverging (correlation > 1)”.

*p<0.05

**p<0.01

***p<0.001
